# Melt Rheology and Mechanical Characteristics of Poly(Lactic Acid)/Alkylated Graphene Oxide Nanocomposites

**DOI:** 10.3390/polym12102402

**Published:** 2020-10-19

**Authors:** In Hye Park, Jae Yoon Lee, Seung Jae Ahn, Hyoung Jin Choi

**Affiliations:** 1 Department of Polymer Science and Engineering, Inha University, Incheon 22212, Korea; fineinpark@gmail.com (I.H.P.); neostar9@naver.com (J.Y.L.); 2 Department of Chemical Engineering, Inha University, Incheon 402-751, Korea; sjahn@kitech.re.kr

**Keywords:** poly(lactic acid), graphene oxide, rheology, alkylation, nanocomposite

## Abstract

Poly(lactic acid) (PLA) nanocomposites were synthesized by a solution blending and coagulation method using alkylated graphene oxide (AGO) as a reinforcing agent. Turbiscan confirmed that the alkylation of GO led to enhanced compatibility between the matrix and the filler. The improved dispersity of the filler resulted in superior interfacial adhesion between the PLA chains and AGO basal plane, leading to enhanced mechanical and rheological properties compared to neat PLA. The tensile strength and elongation at break, i.e., ductility, increased by 38% and 42%, respectively, at the same filler content nanocomposite (PLA/AGO 1 wt %) compared to nonfiller PLA. Rheological analysis of the nanocomposites in the molten state of the samples was performed to understand the filler network formed inside the matrix. The storage modulus increased significantly from PLA/AGO 0.5 wt % (9.6 Pa) to PLA/AGO 1.0 wt % (908 Pa). This indicates a percolation threshold between the two filler contents. A steady shear test was performed to examine the melt flow characteristics of PLA/AGO nanocomposites at 170 °C, and the viscosity was predicted using the Carreau−Yasuda model.

## 1. Introduction

Finding materials derived from natural resources has become an emerging issue to replace the original fossil fuel resources because of recent global environmental problems caused by fossil fuel-based plastics [1,2]. Among the synthetic plastics, poly(lactic acid) (PLA), which can also be obtained from biobased materials, is nontoxic, biodegradable, and biocompatible, and is one of the most well-known biodegradable polymers [3,4]. PLA can be applied in many industries, such as automotive parts, food packaging, textiles, and medical devices [5,6]. On the other hand, its slow crystallization rate, brittleness, and inherent mechanical properties are still insufficient for practical use. Therefore, numerous attempts have been made to improve the physical characteristics of PLA, for example, the addition of a plasticizer (i.e., poly(ethylene glycol) (PEG), tributyl citrate), or a nucleating agent, such as boron nitride, calcium carbonate, and talc [7,8,9,10,11]. The incorporation of a nanofiller to the PLA matrix is a significant solution for the various problems of PLA, such as clay [12], carbon nanotubes (CNTs) [13], cellulose nanocrystals (CNCs) [14], and graphene [15]. Graphene is a two-dimensional multilayered carbon material that has attracted enormous interest because of its large specific area, superior strength, low density, and remarkable electrical and thermal characteristics [16].

The effectiveness of the filler generally comes from the physical cross-linking points between the polymer matrixes. Hence, to achieve the maximum efficiency of the filler, the graphene sheets must be dispersed homogeneously in the polymer matrix with improved interfacial adhesion with the polymer. Unfortunately, the graphene tends to agglomerate due to strong van der Waals interactions and has poor compatibility with many polymers, leading to the poor performance of its composites [17]. Therefore, hydroxyl-associated –OH, –O–(basal plane) and –COOH functionalized graphene, called graphene oxide (GO), has been studied owing to its good dispersity in polar matrices and its ease of functionalization [18,19]. Many studies have examined PLA/GO nanocomposites to enhance the characteristics of PLA, such as crystallization [20], thermal resistance [21], fire resistance [22], and mechanical behavior [23].

Some rheological studies of PLA/GO nanocomposites have recently been reported [24,25], but extensive characterization has seldom been carried out. Rheological studies of nanocomposites are crucial to proving the performance of polymeric nanocomposites derived by the interconnection and dispersion state of the fillers in the polymer matrices in addition to managing their flow characteristics, which are strongly associated with their processability. The rheological properties depend on the filler size, shape, and surface chemistry. Thus, by combining the chemistry, morphology of the filler, and rheological properties of the composite, the tester can obtain the valuable part during a new process setup [26,27].

In this study, alkylated graphene oxide (AGO) was synthesized for use as an effective filler, which was aimed at improving compatibility and interfacial adhesion with the polymer, PLA [28]. With AGO dispersed in the CHCl_3_ solvent, the PLA/AGO nanocomposite was fabricated by solution blending and the widely known coagulation method [29,30]. The AGO could be dispersed homogeneously in the PLA matrix because of its increased hydrophobicity and enhanced interfacial adhesion of the PLA chains on the AGO surface, meaning increased compatibility with PLA. Such effects have led to PLA/AGO nanocomposites with enhanced mechanical strength and toughness. In particular, a 42% increase in tensile strength and a 38% increase in elongation at break was observed at the UTM test of the PLA/AGO (1 wt %) nanocomposite. In addition, dynamic and steady shear rheological tests were addressed in the study. The internal network structure of the filler with the PLA and the relationship with the rheological measurement are discussed.

## 2. Experimental

### 2.1. Materials and Synthesis

The biodegradable PLA (grade 2003D, M_w_ = 157,503 Da) purchased from Nature Works LLC (Minnetonka, Minnesota) had a melting temperature, density, and melt index between 145 to 160 °C, 1.24 g/cc, and 6 g/10 min, respectively. The PLA pellets were kept in a vacuum oven before being used in solution melt blending. Graphene oxide (GO) (grade ABX-ADCP-GO) in the form of acidic 25% aqueous dispersion [31] was purchased from Angstron Materials (Dayton, OH, USA). The 1-Methoxy-2-propanol (called propylene glycol methyl ether) (PGME) and octylamine were acquired from Sigma-Aldrich (St. Louis, MO, USA). Chloroform (CHCl_3_), which is used as a solution blending solvent, was supplied by Daejung Chem. (Busan, South Korea). All products were used without further treatment.

For the alkylation of GO, 200 mg of GO was dispersed in PGME for 30 min. The solution was transferred to a three-neck reactor and set to 80 °C. Octylamine was then added dropwise to the solution and stirred for 24 h. The surface-alkylated GO (AGO) was centrifuged at 4 °C to eliminate the unreacted octylamine. The solvent was changed to CHCl_3_ to make the mother solution of AGO/CHCl_3_ 0.2 wt %.

**Scheme 1:**
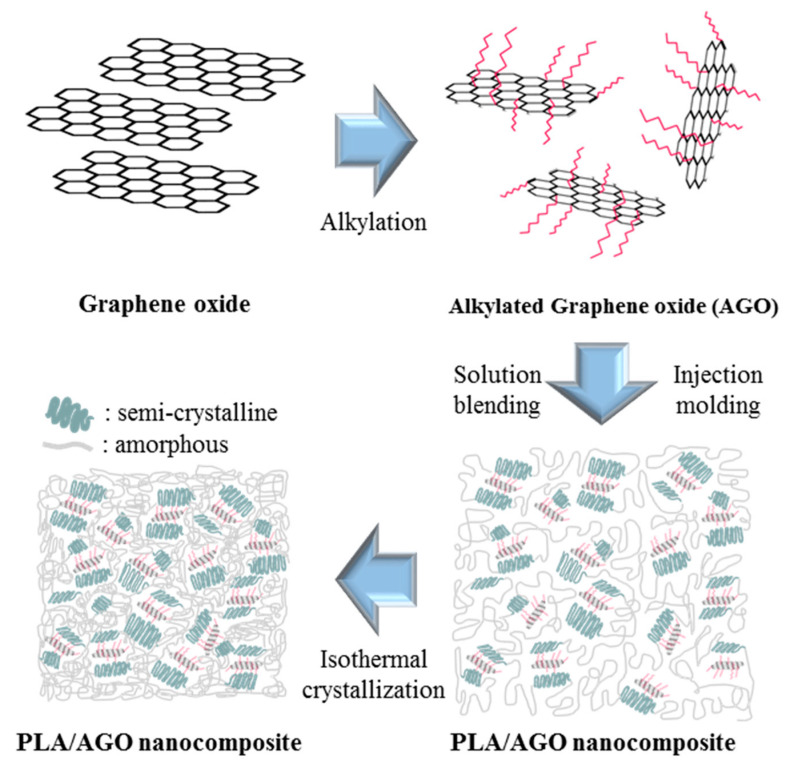
Overall schematic diagram from alkylation of graphene oxide (GO) sheet to processing PLA/AGO nanocomposite.

PLA/AGO nanocomposite was synthesized using the following process. The AGO/CHCl_3_ solution was dispersed in a CHCl_3_ solvent and 5 g of PLA to make the PLA/AGO nanocomposite with diverse contents (neat, 0.1, 0.3, 0.5, 1.0, and 2.0 wt %). The total CHCl_3_ weight was fixed to 100 g. Solution blending was performed at 45 °C until the PLA and AGO were blended homogeneously. After blending, the PLA/AGO composite was precipitated in methanol using a coagulation method [29]. The resulting composite was dried in a vacuum oven at 30 °C for three days to eliminate the remaining CHCl_3_ and methanol. The same procedure was performed using the GO filler, making PLA/GO 1 wt % composite for comparison. Scheme 1 presents the overall experimental procedure.

The alkylation of GO was performed by adding octylamine to the dispersion of GO and PGME. The amine group reacted with the epoxide group via an S_N_2 reaction, which was positioned at the basal plane of GO. After the reaction, the AGO dispersion was washed several times by centrifugation. The next step was the solution blending of the filler (AGO) and polymer resin (PLA). Five grams of PLA and a certain amount of AGO were dispersed in CHCl_3_ at 45 °C. After one hour, a mixture of thread-like precipitate was obtained by the coagulation method using methanol. The PLA/AGO was spread and dried in a vacuum oven for three days to eliminate the remaining CHCl_3_ solvent. PLA/AGO nanocomposites with different contents of AGO filler were injection molded using a minimax molder. Subsequently, an isothermal crystallization process was performed at 70 °C for one hour.

### 2.2. Characterization

The dispersion stability of AGO in CHCl_3_ solvent was analyzed using a Turbiscan (Classic MA2000, Formulaction, Toulouse, France). The equipment was used for two days at room temperature. Before the solution was placed into the measurement cell, both GO and AGO were blended with CHCl_3_ by 0.2 wt %, homogenized for 3 min. The alkylated functional group was confirmed by Fourier transform infrared (FT-IR) (VERTEX 80 V, Bruker, Ettlingen, Germany) spectroscopy. GO and AGO powder were ground with KBr powder at a 1:200 weight ratio and pelletized to a disk-type sample. The pellet was dried in a vacuum oven for one day. The morphology of the PLA/AGO nanocomposite with different contents of AGO filler was analyzed by transmission electron microscopy (TEM) (Philips CM200, Eindhoven, The Netherlands). The sample was cut into 100 nm thick pieces via ultra-microtoming. The microtomed samples were floated on the water and then put on the copper grid using tweezers. They were finally dried in a dry oven before TEM testing. Differential scanning calorimetry (DSC) (DSC 200F3, NETZSCH, Selb, Germany) was carried out in a nitrogen atmosphere. Both temperature and heat flow were calibrated using indium. The DSC trace cycle was acquired by heating and cooling (two cycles) at a heating rate of 10 °C/min from 25 to 210 °C. X-ray diffraction (Pro MRD, Malvern Panalytical Ltd., Eindhoven, The Netherlands) was conducted over the scan range of 5°−60° 2θ at a scan rate of 1°/min using Cu Ka radiation. The dried PLA/AGO blend was processed using an injection molding machine (Minimax molder, LabCamp, Paju, Korea) under 90 rpm at 190 °C, and ASTM D638 Type 5 dog-bone specimens were received. Universal tensile testing (Instron 5569, Instron, Norwood, MA, USA) was performed at 25 °C with an extension rate of 1 mm/min and a load cell of 1 kN and a gauge length of 10 mm. Viscoelasticity and flow properties of the PLA/AGO nanocomposites were tested using a rotational rheometer (MCR 102, Anton-Paar, Graz, Austria) at 170 °C with a parallel-plate (PP20) device. The steady, simple shear measurement was plotted as the shear stress versus shear rate from 0.01 to 1 s^−1^. In the dynamic oscillatory shear experiment, the strain was fixed to 0.01, and the angular frequency was varied from 0.1 to 500 rad/s.

## 3. Results and Discussions

### Material Property

**Figure 1:**
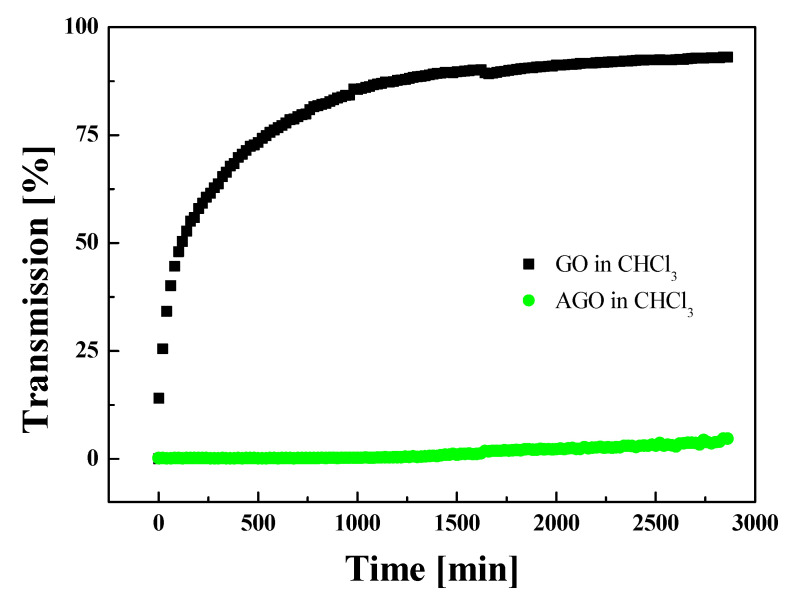
Change in transmission (%) as a function of time of GO (solid), AGO (dot) in CHCl_3_ solution (0.2 wt %).

Turbiscan, which detects the light transmittance through the sample solution, was used for two days to compare the dispersion stability of AGO and GO in the CHCl_3_ solvent. The transmittance was converted to the sedimentation ratio, as plotted in Figure 1. The AGO solution merely settled (3.8% transmission at 2800 min) compared to the GO solution (93% transmission at 2800 min). This was attributed to the different surface properties of GO and AGO. The alkylated surface of AGO makes the graphene sheet more hydrophobic, and the alkyl chain protects the graphene sheet to prevent agglomeration. On the other hand, GO agglomerates owing to the strong, attractive interactions (i.e., van der Waals and hydrogen bonding), causing flocculation and disturbance of the dispersion stability of the solution. Moreover, the mere settlement of AGO can be an indirect message that AGO has a similar solubility parameter with CHCl_3_, which will have a positive effect when solution blending with PLA, and which confirms CHCl_3_ as a good solvent.

**Figure 2:**
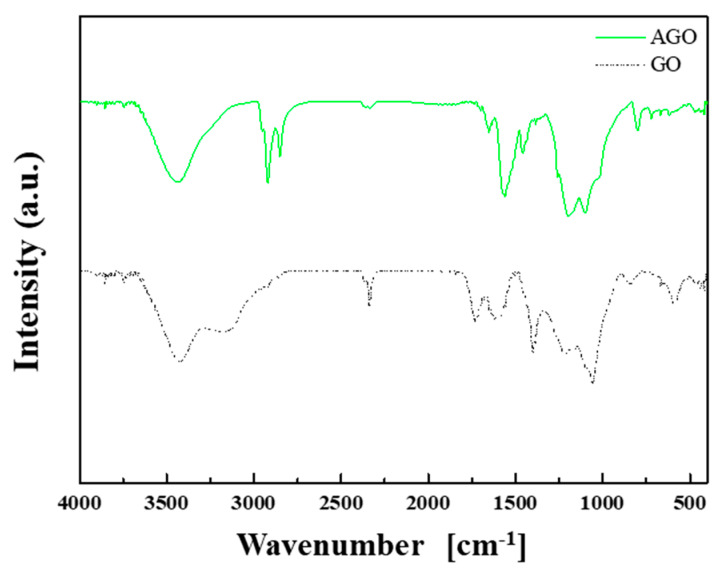
FT-IR spectrum of AGO (solid line) and GO (dotted line).

Alkylation of the GO surface was also estimated by FT-IR spectroscopy from 400 to 4000 cm^−1^. Figure 2 shows the FT-IR spectrum of AGO and GO as a solid and dotted line, respectively. The IR characteristic peaks of GO were observed at 1733 cm^−1^ (C=O stretching vibration), 1610 cm^−1^ (aromatic ring C=C), 1398 cm^−1^ (carboxyl C−OH stretching), and 1058 cm^−1^ (alkoxy C−O stretches) [32]. In addition, the wideband at 3450 cm^−1^ was attributed to hydroxyl groups. On the other hand, new peaks for the CH_2_ and CH_3_ stretching vibrations in 2920 and 2850 cm^−1^, respectively, were observed when the octyl chain was substituted on the GO surface. Hence, the alkyl chain has been substituted successfully on the graphene sheet instead of the carboxylic group and epoxy group.

**Figure 3:**
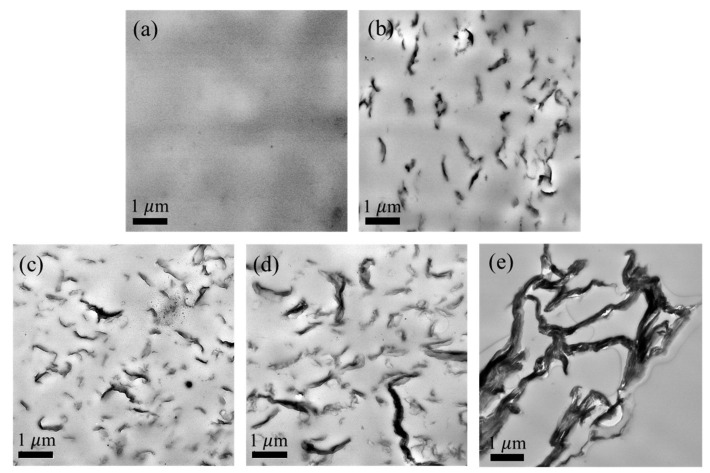
TEM images of (**a**) neat PLA and PLA/AGO nanocomposites (**b**) 0.5 wt %, (**c**) 1.0 wt %, (**d**) 2.0 wt % and (**e**) PLA/GO 1 wt %.

The morphology and dispersity of AGO in the PLA matrix were observed by TEM. Figure 3 presents an image of the PLA/AGO nanocomposites with different filler ratios and comparison images of neat PLA (a) and PLA/GO (e) composite. A comparison of Figure 3c,e showed that AGO was in a superior dispersed state to the unmodified GO in the PLA matrix. In Figure 3c, the AGO was dispersed as nanosized particles, and there was virtually no clear space. In Figure 3e, however, GO was aggregated due to the attraction force between the filler itself, and the clear space comprised a large portion of the composite.

**Figure 4:**
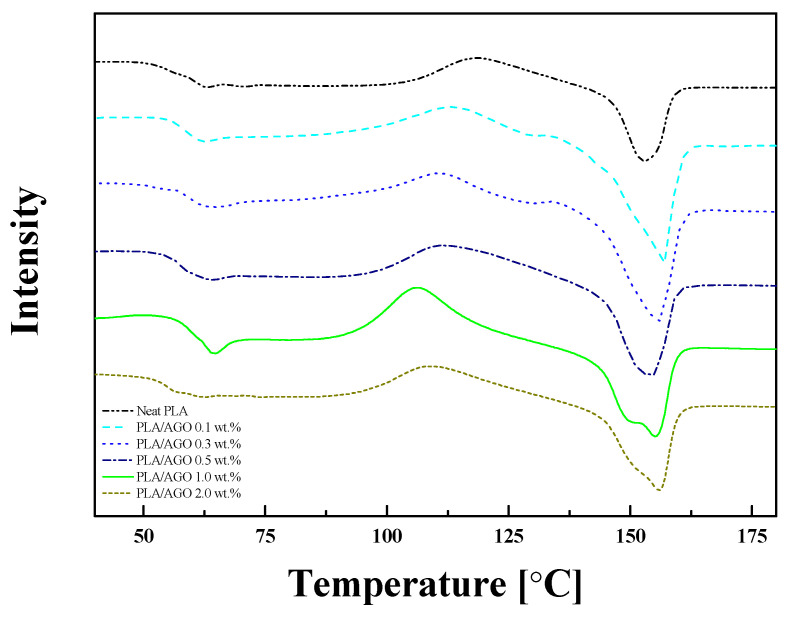
Differential scanning calorimetry (DSC) heating curves of pristine PLA and PLA/AGO nanocomposites of different weight ratios.

DSC measurements of the contents of the PLA/AGO composites were taken to analyze the crystallization behavior. Figure 4 plots the endo (down)—exo (up) curve at a heating rate of 10 °C/min, and Table 1 lists the characteristic thermal peaks. The degree of crystallinity (χ) was calculated using the following equation [33,34]:

(1) $$\chi =\frac{\Delta H_{m}}{\Delta H_{m}^{\infty }\omega }\times 100\;\left[\%\right]$$

where $\Delta H_{m}^{\infty }$ is the heat of fusion (=93 J/g) of completely crystalline PLA [35]. Three significant changes highlighted the positive effect of the crystallinity of the PLA/AGO nanocomposites due to the addition of AGO: increase in glass transition temperature (T_g_), decrease in cold crystallization temperature (T_cc_), and the gradual peak-splitting phenomena of melting temperature (T_m_) with increasing AGO content. As listed in Table 1, the T_g_ of the PLA/AGO composites was higher than that of the neat PLA. Note that T_g_ is a temperature showing the absorbed energy for the reptile motion of the polymeric chain. The incorporation of an AGO filler might disturb the glassy movement of the polymer chains due to the affinity between the filler with the polymer matrix, which requires considerably more energy, leading to an increase in T_g_ [36,37]. Despite our effort to estimate them with as little uncertainty as possible with the increase of AGO content, especially in the case of 2 wt %, two potential T_g_s could be estimated because of aggregated AGO in the nanocomposite and we chose the lower one. In addition, related to the T_g_, the reason why the T_g_ increased until the percolation threshold and then decreased at 2 wt % (similar with neat PLA) is as follows. At first, the compatibility between the polymer matrix (PLA) and filler (AGO) could increase the T_g_, in which the similar surface energy actually leads to the increase in the interaction between the matrix and the filler. Secondly, we can consider the optimum conditions for the nanoconfinement effect. It could be regarded that due to aggregation of the AGO filler in PLA/AGO 2 wt %, the particle size of the AGO increased, hindering the confinement of the PLA chain. This is the reason why the T_g_ of PLA/AGO 2 wt % decreased and did not increase compared with the neat PLA. T_cc_ decreased with the incorporation of AGO, which was also observed by other groups [35,38]. This was attributed to the enhanced crystallization behavior through the incorporation of AGO, which supplied heterogeneous nucleation sites to the PLA/AGO nanocomposites [39]. The gradual appearance of two peaks in T_m_ could be further evidence that AGO acts as a nucleating agent. At the early T_m1_, the crystal formed at the outer layer of AGO was degraded. T_m2_ was the temperature peak for the degradation of the crystal formed at the surface of AGO [35].

**Figure 5:**
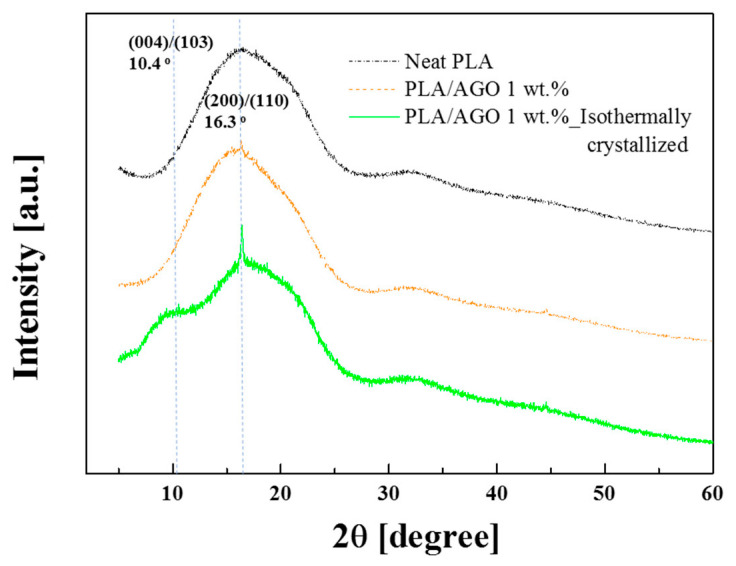
The 1D-XRD curves showing crystal growth in the isothermal crystallization of PLA/AGO nanocomposite (solid) compared with untreated PLA/AGO nanocomposite (dash) and neat PLA (dash-dot).

Despite its potential applications, such as the biomedical and packaging industries, PLA still lacks the appropriate mechanical properties for injection and extrusion molding [40]. Crystallinity plays a crucial role in controlling mechanical performance in rigid molded applications [41]. This study compared the crystallization behaviors of isothermal crystallized PLA/AGO nanocomposites, neat PLA, and PLA/AGO 1 wt % without any treatment by XRD (Figure 5). First, by comparing the neat PLA and untreated PLA/AGO 1 wt %, a slight increase in the (200)/(110) α′ form of the PLA homocrystallite was formed at 16.3° 2θ [42,43]. After isothermal crystallization at 70 °C, a significant increase was observed at the same spot (16.3° 2θ). Moreover, a shoulder peak was observed at 10.4° 2θ, which is also a sign of the (004)/(103) α form of PLA [44]. Therefore, incorporating an AGO filler and isothermal crystallization promotes nucleation and crystal growth.

**Figure 6:**
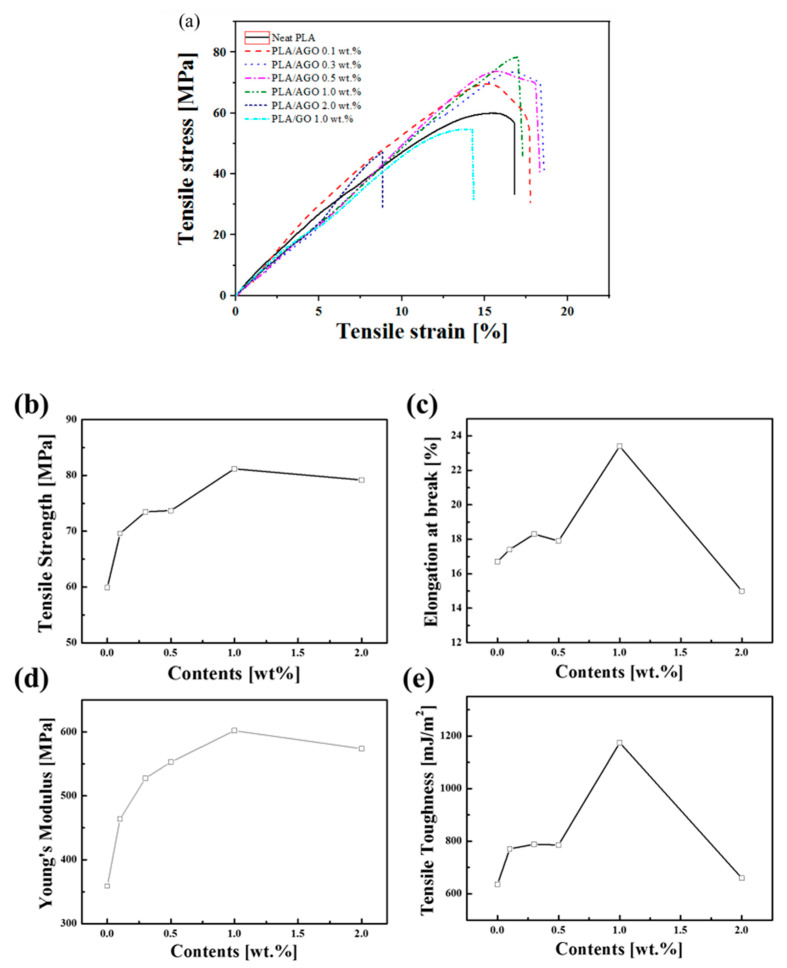
(**a**) Stress−strain curves of pure PLA and PLA/AGO composites and variation of (**b**) tensile strength (**c**) elongation at break (**d**) Young’s modulus (**e**) tensile toughness as a function of the AGO contents.

A tensile test was performed using a universal tensile testing machine (UTM) with an ASTM D638 Type 5 dog-bone specimen to examine the mechanical properties according to the crystallization characteristics and homogeneous dispersion of AGO. Figure 6a presents the typical stress−strain curve of PLA/AGO nanocomposites, neat PLA, and PLA/GO. The PLA/GO composite showed inferior performance compared to the PLA/AGO nanocomposites, which is in accordance with the poor dispersity of agglomerated GO, as shown in Figure 2e. The typical properties derived from the stress−strain plot, such as the tensile strength, elongation at break, Young’s modulus, and tensile toughness, are given as a function of the filler contents, as shown in Figure 6b–d. In Figure 6b,d, the tensile strength and Young’s modulus increased with the addition of AGO until 1 wt %. The tensile strength of PLA/AGO 1 wt % (81.2 MPa) was 37.8% higher than that of neat PLA (59 MPa) and slightly lower than that of PLA/AGO 2 wt %. The Young’s modulus also showed similar behavior: the value increased from 358 MPa to 602 MPa and decreased above 1 wt %. This effect arose from the higher mechanical properties that AGO possesses compared to PLA. The homogeneously dispersed AGO might act as an effective load-transfer medium. Generally, the stiffness in polymeric composites has an inverse relationship with the ductility [45,46]. Therefore, it is easy to see data with an inverse tendency between tensile strength and the elongation at break. On the other hand, as shown in Figure 6c, the elongation at break also reached a maximum at PLA/AGO 1 wt %, which was 42% higher than that of neat PLA along with the maximum tensile toughness (1174.9 mJ/m^2^), as shown in Figure 6e. Standard deviation of all these mechanical characteristic values was below 5%. This remarkable property originates from interfacial adhesion between PLA and AGO [47]. The strong adhesion and interaction can prevent the PLA/AGO nanocomposites from crack propagation and enhance load transfer during the tensile test [37].

**Figure 7:**
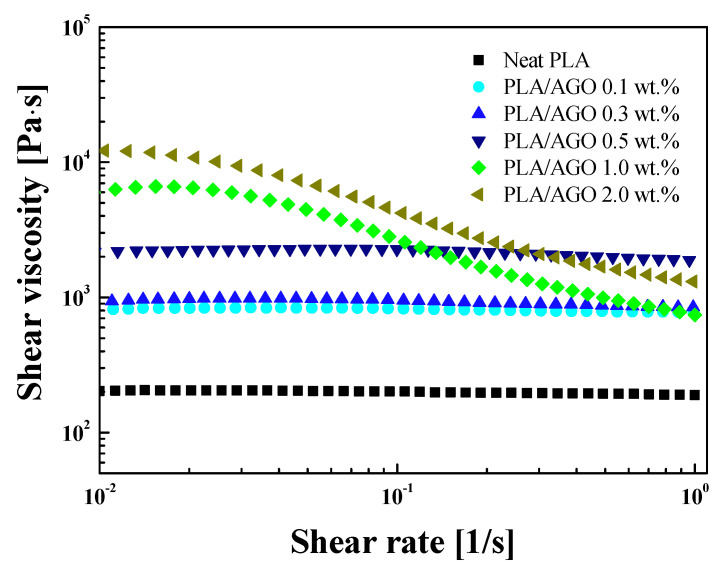
Shear viscosity curves of PLA/AGO nanocomposites at a constant temperature.

The flow behavior of PLA/AGO nanocomposites with different AGO contents and neat PLA was analyzed using a rotational rheometer MCR102, as shown in Figure 7. The controlled shear rate (CSR) test was performed over the shear rate range of 0.01 to 1 s^−1^ at 170 °C. As shown in the figure, the initial viscosity increased with increasing filler content, especially from 0.5 wt % to 1.0 wt %. The neat PLA exhibited Newtonian behavior of a constant shear viscosity, which is independent of the shear rate. On the other hand, the sample with high filler content exhibited non-Newtonian behavior. For PLA/AGO 1.0 and 2.0 wt %, shear-thinning of the viscosity was detected compared to the lower filler contents. In the case of PLA/AGO 0.1 and 0.3 wt %, the shear viscosity behavior was similar to the neat PLA. This was attributed to the internal networking structure of the filler, which will also be explained in the oscillatory shear experiment. The flow curve of the PLA/AGO samples could be fitted using the Carreau-Yasuda equation [48,49]:

(2) $$\frac{\eta -\eta_{\infty }}{\eta_{0}-\eta_{\infty }}=\frac{1}{{\left[1+{\left(\lambda \dot{\gamma }\right)}^{a}\right]}^{\left(1-n\right)/a}}$$

where $\eta_{0}$ and $\eta_{\infty }$ are the shear viscosity of zero and infinite shear rate, respectively. *λ* [s] is the relaxation time; n is the dimensionless parameter (0 ≤ *n* < 1), and a is the parameter that depicts the transition zone from Newtonian to the shear-thinning region. Table 2 lists the calculated parameters.

The characteristic time *λ* was significantly large at higher filler contents, which were 1.0 and 2.0 wt %. This indicates early termination of the pseudo-solid property during the low shear rates and higher rigidity at high AGO contents.

**Figure 8:**
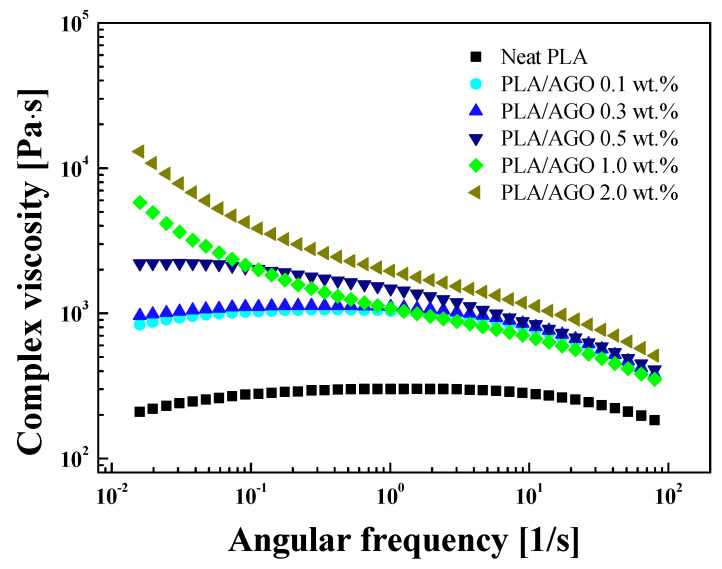
Complex viscosity as a function of the frequency of the PLA/AGO nanocomposite in the dynamic oscillation model.

From the dynamic oscillatory shear measurements, the complex viscosity was obtained as a function of the frequency, as shown in Figure 8. The complex viscosity showed a similar trend to that of the shear viscosity and exhibited a strong dependency on the addition of AGO filler. Neat PLA showed a Newtonian plateau in the low angular frequency region. As the filler content increased, however, the composite showed a shear-thinning property. The initial complex viscosity increased with increasing AGO, and showed a typical sharp increase in the filler content between PLA/AGO 0.5 wt % (1990 Pa∙s) and PLA/AGO 1.0 wt % (5850 Pa∙s). Such behavior was attributed to the change in the internal microstructure of the filler, which is expected to be cross-linked because the filler content is above the percolation threshold [50].

**Figure 9:**
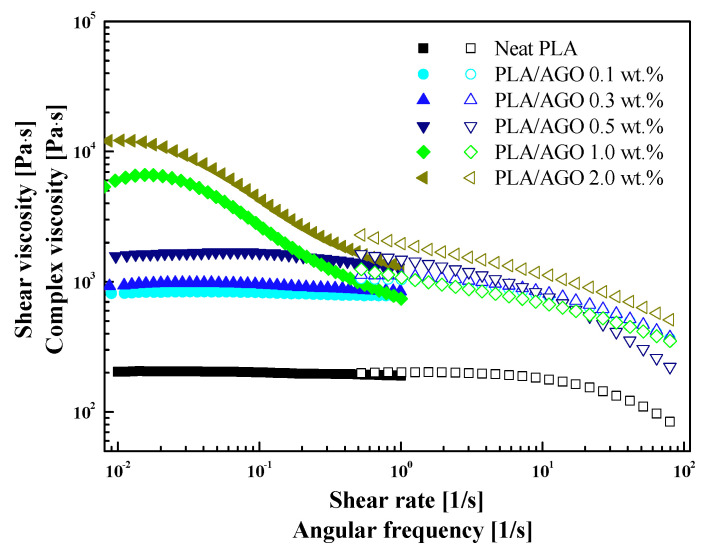
Cox–Merz relationship of shear (closed symbol) and complex (open symbol) viscosities.

In the CSR mode test, rheological data at a very high shear rate level was difficult to obtain with a parallel-plate geometry. The Cox−Merz rule, which states that the shear viscosity *η* and complex viscosity $\eta^{*}$ are equivalent when compared at the same unit as [s^−1^] as shown in Figure 9. The relationship between η and $\eta^{*}$ can be described as follows [51]:

(3) $${\eta \left(\dot{\gamma }\right)|}_{\dot{\gamma }=\omega }=\left|\eta^{*}\left(\omega \right)\right|=\sqrt{\eta^{\prime }{}^{2}\left(\omega \right)+\eta^{\prime \prime }{}^{2}}$$

**Figure 10:**
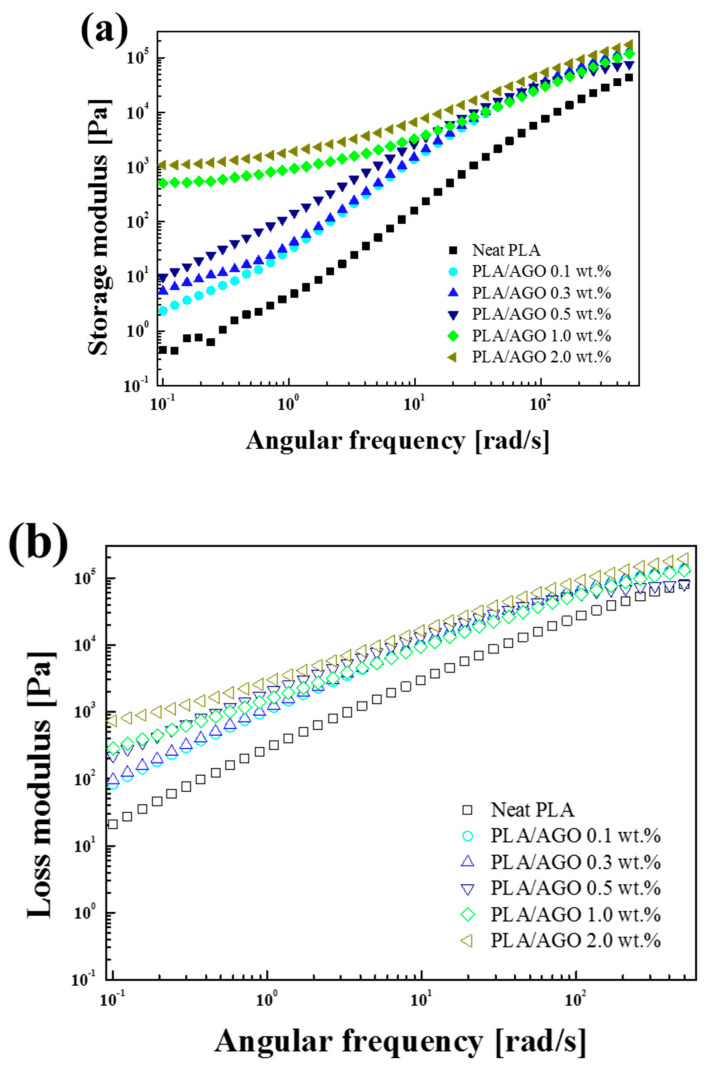
Frequency sweep of the PLA/AGO nanocomposites at a fixed strain (0.01) chosen in their linear viscoelastic region. (**a**) storage modulus; (**b**) loss modulus.

Figure 10 presents the storage ($G^{\prime }$) and loss ($G^{\prime \prime }$) modulus of the PLA/AGO nanocomposites as a function of frequency. The graph shows the effect of incorporating AGO filler on the rheological property. The difference was observed more clearly in the low-frequency region. Both $G^{\prime }$ and $G^{\prime \prime }$ of PLA/AGO nanocomposite presented in Figure 10a and Figure 10b, respectively increased with increasing AGO loading and the dependence on the frequency decreased significantly at high AGO contents (1.0 and 2.0 wt %). The formation of a $G^{\prime }$ plateau indicates the 3-D percolation network of AGO in the PLA matrix, which is stated as a nonterminal behavior [15,52,53]. Such behavior is in accordance with the result of complex viscosity, in which shear-thinning occurred abruptly from 1.0 wt %. In the low-frequency region, the low filler content nanocomposites (neat, 0.1–0.5 wt %) showed terminal behavior. In particular, the neat PLA had a slope of ~2. In the literature, the homo-dispersed polymer chains follow the power−law relation of $G^{\prime }∼\omega^{2}$ in the terminal flow behavior. The incorporation of AGO gradually develops the filler network and limits the motion of the PLA chains, resulting in the decreased dependency of $G^{\prime }$ to the frequency and increased $G^{\prime }$ value in the low-frequency region [54].

**Figure 11:**
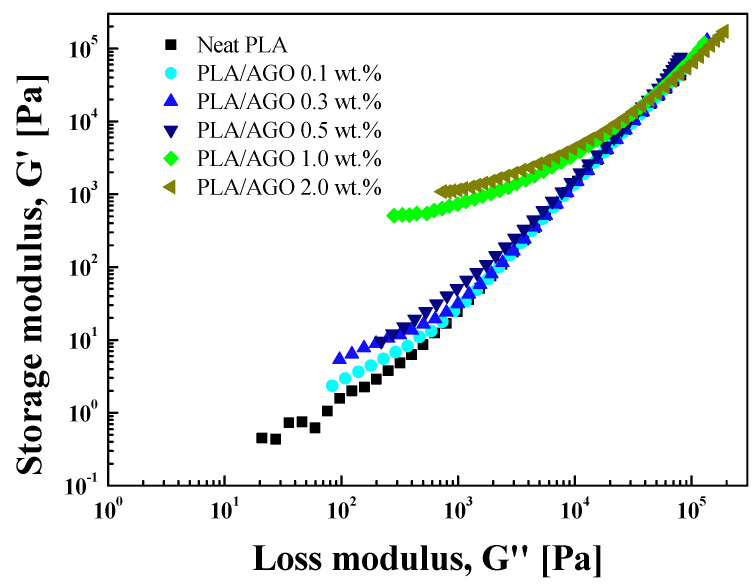
Modified Cole−Cole plot of the PLA/AGO nanocomposites.

Figure 11 shows the modified Cole−Cole curve, which can also explain the effects of incorporating fillers on the structural changes of the composite [55,56]. Because the rheological and processing characteristics change abruptly above the percolation threshold of the nanofiller, the relationship between $G^{\prime }$ and $G^{\prime }$ changes dramatically by the formation of a network structure in the filler. In the figure, low AGO loading samples (0.1 to 0.5 wt %) have a similar tendency to neat PLA, whereas the slope decreases for high AGO filler loading above 1.0 wt %, which indicates a change in the microstructure of the AGO filler.

**Figure 12:**
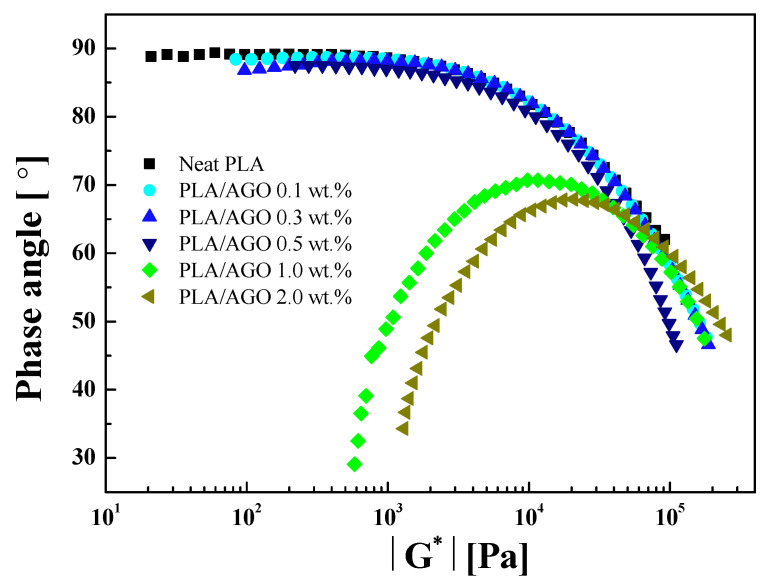
Van Gurp−Palmen plot of five samples of PLA/AGO nanocomposites and neat PLA.

In addition to the modified Cole−Cole plot, the formation of a solid-like structure can also be explained by the van Gurp−Palmen (VGP) plot in Figure 12. The VGP plot is a diagram of the phase angle in the function of the complex modulus. The low loading of the AGO nanocomposites (0.1–0.5 wt %) showed similar behavior to the neat PLA. In particular, the composite showed a viscous property for a phase angle close to 90° in a low complex modulus region. For the samples with a high loading AGO, however, the values were below 45° in a low complex modulus region. The dramatic decrease in the phase angle indicated a physically cross-linked network structure of AGO fillers [57,58].

## 4. Conclusions

The alkylation of GO was performed by grafting octylamine onto the surface of GO. Octylamine reacted with the –OH, –O–(basal plane), and –COOH functionalities of GO, providing hydrophobicity and a layer-to-layer distance of the graphene sheet. Such properties are the key to inhibiting the agglomeration of AGO filler itself and increasing the compatibility with the PLA matrix. FT-IR and turbiscan confirmed the presence of the alkyl chains on the GO surface. The alkane C-H chain peak increased abruptly according to the FT-IR spectra, which led to longer dispersion stability in CHCl_3_, which was confirmed by Turbiscan. Crystallization studies using DSC showed that the incorporation of AGO facilitated the crystallization process of PLA, and the crystallinity was higher than that of the neat PLA. UTM analysis revealed a significant increase in the mechanical properties of the PLA/AGO nanocomposite. By incorporating 1 wt % AGO to the PLA, the G’ increased up to 35% compared to neat PLA, and the elongation at break increased to 42%. This was attributed to the superior compatibility between AGO and the PLA matrix, which enhanced the interfacial adhesion between the filler and the matrix. The rheological measurements also confirmed a filler network in the PLA matrix, showing a percolation threshold between the filler contents of 0.5 and 1.0 wt %. The modulus and viscosity increased as more AGO was added to the PLA, and the viscosity was fitted to the predicted model. On the other hand, we anticipate that the biodegradable, ecofriendly PLA and its nanocomposites could replace petrochemical-based materials in various engineering fields, including food packaging and medical devices. Especially, the PLA/AGO nanocomposite could increase the barrier characteristics against gas and water vapor transfer for packaging.

## Figures and Tables

**Table 1:** Characteristic temperatures obtained from DSC of the PLA/AGO nanocomposites.

Samples	T_g_ [°C]	T_cc_ [°C]	T_m_ [°C]		$\Delta H_{m}$ [J/g]	χ [%]
			T_m1_	T_m2_		
Neat PLA	56.5	117.9	-	152.8	15.44	19.77
PLA/AGO 0.1	58.6	113.2	-	156.4	29.1	30.34
PLA/AGO 0.3	58.9	110.3	-	155.1	30.36	31.72
PLA/AGO 0.5	57.2	111.2	-	154.2	24.13	25.39
PLA/AGO 1.0	59.8	106.2	149.8	155	23.15	24.23
PLA/AGO 2.0	55.1	108.9	150.8	156	20.93	22.25

**Table 2:** Carreau−Yasuda model parameters of viscosity fitting.

Sample	$\eta_{0}$	Λ	*n*	$\eta_{\infty }$	R^2^
Neat	203	6.8	0.3656	186	0.93
PLA/AGO 0.1	833	5.627	0.1032	764	0.96
PLA/AGO 0.3	976	6.186	0.4511	823	0.98
PLA/AGO 0.5	2290	0.7193	0.1874	1120	0.99
PLA/AGO 1.0	6610	22.86	0.2123	374	0.99
PLA/AGO 2.0	12,200	29.89	0.1002	834	0.99

## References

[1] Chen C.C., Chueh J.Y., Tseng H., Huang H.M., Lee S.Y. (2003). Preparation and characterization of biodegradable PLA polymeric blends. Biomaterials.

[2] Ruiz-Mercado G.J., Smith R.L., Gonzalez M.A. (2012). Sustainability indicators for chemical processes: II Data needs. Ind. Eng. Chem. Res..

[3] Bourbigot S., Fontaine G. (2010). Flame retardancy of polylactide: An overview. Polym. Chem..

[4] Narimissa E., Gupta R.K., Choi H.J., Kao N., Jollands M. (2012). Morphological, Mechanical, and Thermal Characterization of Biopolymer Composites Based on Polylactide and Nanographite Platelets. Polym. Compos..

[5] Harris A.M., Lee E.C. (2013). Durability of polylactide-based polymer blends for injection-molded applications. J. Appl. Polym. Sci..

[6] Xiao L., Wang B., Yang G., Gauthier M. (2012). Poly(lactic acid)-based biomaterials: Synthesis, modification and applications. Biomed. Sci. Eng. Technol..

[7] Li Z., Tan B.H., Lin T., He C. (2016). Recent advances in stereocomplexation of enantiomeric PLA-based copolymers and applications. Prog. Polym. Sci..

[8] Mustapa I.R., Shanks R.A., Daud N. (2018). Morphological structure and thermomechanical properties of hemp fibre reinforced poly(lactic acid) Nanocomposites plasticized with tributyl citrate. Mater. Today Proc..

[9] Mosanenzadeh S.G., Khalid S., Cui Y., Naguib H.E. (2016). High thermally conductive PLA based composites with tailored hybrid network of hexagonal boron nitride and graphene nanoplatelets. Polym. Compos..

[10] Piekarska K., Piorkowska E., Bojda J. (2017). The influence of matrix crystallinity, filler grain size and modification on properties of PLA/calcium carbonate composites. Polym. Test..

[11] Aversa C., Barletta M., Pizzi E., Puopolo M., Vesco S. (2017). Wear resistance of injection moulded PLA-talc engineered bio-composites: Effect of material design, thermal history and shear stresses during melt processing. Wear.

[12] Keshtkar M., Nofar M., Park C.B., Carreau P.J. (2014). Extruded PLA/clay nanocomposite foams blown with supercritical CO_2_. Polymer.

[13] Kuan C.F., Kuan H.C., Ma C.C.M., Chen C.H. (2008). Mechanical and electrical properties of multi-wall carbon nanotube/poly(lactic acid) composites. J. Phys. Chem. Solids.

[14] Dhar P., Bhasney S.M., Kumar A., Katiyar V. (2016). Acid functionalized cellulose nanocrystals and its effect on mechanical, thermal, crystallization and surfaces properties of poly(lactic acid) bionanocomposites films: A comprehensive study. Polymer.

[15] Sabzi M., Jiang L., Liu F., Ghasemi I., Atai M. (2013). Graphene nanoplatelets as poly(lactic acid) modifier: Linear rheological behavior and electrical conductivity. J. Mater. Chem. A.

[16] Zhu Y., Murali S., Cai W., Li X., Suk J.W., Potts J.R., Ruoff R.S. (2010). Graphene and graphene oxide: Synthesis, properties, and applications. Adv. Mater..

[17] Potts J.R., Dreyer D.R., Bielawski C.W., Ruoff R.S. (2011). Graphene-based polymer nanocomposites. Polymer.

[18] Zhang W.L., Park B.J., Choi H.J. (2010). Colloidal graphene oxide/polyaniline nanocomposite and its electrorheology. Chem. Commun..

[19] Zhang W.L., Choi H.J. (2014). Graphene oxide based smart fluids. Soft Matter.

[20] Xu H., Wu D., Yang X., Xie L., Hakkarainen M. (2015). Thermostable and impermeable “nano-barrier walls” constructed by poly(lactic acid) stereocomplex crystal decorated graphene oxide nanosheets. Macromolecules.

[21] Xu H., Feng Z.X., Xie L., Hakkarainen M. (2016). Graphene oxide-driven design of strong and flexible biopolymer barrier films: From smart crystallization control to affordable engineering. Sustain. Chem. Eng..

[22] Bao C., Song L., Xing W., Yuan B., Wilkie C.A., Huang J., Guo Y., Hu Y. (2012). Preparation of graphene by pressurized oxidation and multiplex reduction and its polymer nanocomposites by masterbatch-based melt blending. J. Mater. Chem..

[23] Chen P., Wang Y., Wei T., Meng Z., Jia X., Xi K. (2013). Greatly enhanced mechanical properties and heat distortion resistance of poly(L-lactic acid) upon compositing with functionalized reduced graphene oxide. J. Mater. Chem. A.

[24] Yang L., Zhen W. (2019). Poly(lactic acid)/p-phenylenediamine functionalized graphene oxidized nanocomposites: Preparation, rheological behavior and biodegradability. Eur. Polym. J..

[25] Kang Y., Wang C., Shi X., Zhang G., Chen P., Wang J. (2018). Crystallization, rheology behavior, and antibacterial application of graphene oxide-graft-poly(L-lactide)/poly(L-lactide) nanocomposites. Appl. Surf. Sci..

[26] Xu Z., Niu Y., Yang L., Xie W., Li H., Gan Z., Wang Z. (2010). Morphology, rheology and crystallization behavior of polylactide composites prepared through addition of five-armed star polylactide grafted multiwalled carbon nanotubes. Polymer.

[27] Bhatia A., Gupta R.K., Bhattacharya S.N., Choi H.J. (2009). An investigation of melt rheology and thermal stability of poly(lactic acid)/poly(butylene succinate) nanocomposites. J. Appl. Polym. Sci..

[28] Compton O.C., Dikin D.A., Putz K.W., Brinson L.C., Nguyen S.T. (2010). Electrically conductive “alkylated” graphene paper via chemical reduction of amine-functionalized graphene oxide paper. Adv. Mater..

[29] Kim H., Kobayashi S., AbdurRahim M.A., Zhang M.J., Khusainova A., Hillmyer M.A., Abdala A.A., Macosko C.W. (2011). Graphene/polyethylene nanocomposites: Effect of polyethylene functionalization and blending methods. Polymer.

[30] Gong L., Yin B., Li L.P., Yang M.B. (2015). Nylon-6/Graphene composites modified through polymeric modification of graphene. Compos. B Eng..

[31] The Graphene Catalog: Your Source for Graphene Materials. https://catalog.graphene-info.com/view/30/ABX-ADCP-GO.

[32] Zhang H., Hines D., Akins D.L. (2014). Synthesis of a nanocomposite composed of reduced graphene oxide and gold nanoparticles. Dalton Trans..

[33] Gwon J.G., Cho H.J., Chun S.J., Lee S., Wu Q., Li M.C., Lee S.Y. (2016). Mechanical and thermal properties of toluene diisocyanate-modified cellulose nanocrystal nanocomposites using semi-crystalline poly(lactic acid) as a base matrix. RSC Adv..

[34] Martin O., Avérous L. (2001). Poly(lactic acid): Plasticization and properties of biodegradable multiphase systems. Polymer.

[35] Zhang L., Li Y., Wang H., Qiao Y., Chen J., Cao S. (2015). Strong and ductile poly(lactic acid) nanocomposite films reinforced with alkylated graphene nanosheets. Chem. Eng. J..

[36] Oh H., Green P.F. (2009). Polymer chain dynamics and glass transition in athermal polymer/nanoparticle mixtures. Nat. Mater..

[37] Choe J.H., Jeon J., Lee M.E., Wie J.J., Jin H.J., Yun Y.S. (2018). Nanoconfinement effects of chemically reduced graphene oxide nanoribbons on poly(vinyl chloride). Nanoscale.

[38] Feng Y., Ma P., Xu P., Wang R., Dong W., Chen M., Joziasse C. (2018). The crystallization behavior of poly (lactic acid) with different types of nucleating agents. Int. J. Biol. Macromol..

[39] Tang H., Chen J.B., Wang Y., Xu J.Z., Hsiao B.S., Zhong G.J., Li Z.M. (2012). Shear flow and carbon nanotubes synergistically induced nonisothermal crystallization of poly(lactic acid) and its application in injection molding. Biomacromolecules.

[40] Harris A.M., Lee E.C. (2008). Improving mechanical performance of injection molded PLA by controlling crystallinity. J. Appl. Polym. Sci..

[41] Bai H., Huang C., Xiu H., Zhang Q., Fu Q. (2014). Enhancing mechanical performance of polylactide by tailoring crystal morphology and lamellae orientation with the aid of nucleating agent. Polymer.

[42] Sun Y., He C. (2012). Synthesis and stereocomplex crystallization of poly(lactide)–graphene oxide nanocomposites. ACS Macro Lett..

[43] Zhang J., Tashiro K., Tsuji H., Domb A.J. (2007). Investigation of phase transitional behavior of poly (L-lactide)/poly (D-lactide) blend used to prepare the highly-oriented stereocomplex. Macromolecules.

[44] Xu J.Z., Zhang Z.J., Xu H., Chen J.B., Ran R., Li Z.M. (2015). Highly enhanced crystallization kinetics of poly(l-lactic acid) by poly(ethylene glycol) grafted graphene oxide simultaneously as heterogeneous nucleation agent and chain mobility promoter. Macromolecules.

[45] Chartarrayawadee W., Molloy R., Ratchawet A., Janmee N., Butsamran M., Panpai K. (2017). Fabrication of poly(lactic acid)/graphene oxide/stearic acid composites with improved tensile strength. Polym. Compos..

[46] Qian S., Sheng K. (2017). PLA toughened by bamboo cellulose nanowhiskers: Role of silane compatibilization on the PLA bionanocomposite properties. Compos. Sci. Technol..

[47] Lu H., Chen Z., Ma C. (2012). Bioinspired approaches for optimizing the strength and toughness of graphene-based polymer nanocomposites. J. Mater. Chem. C.

[48] Narimissa E., Gupta R.K., Kao N., Choi H.J., Jollands M., Bhattacharya S.N. (2014). Melt rheological investigation of polylactide-nanographite platelets biopolymer composites. Polym. Eng. Sci..

[49] Das D., Sethy S., Satapathy B.K. (2018). Matrix tacticity controlled tuning of microstructure, constitutive behavior and rheological percolation effect of melt-mixed amino-functionalized MWCNT/PP nanocomposites. Polym. Eng. Sci..

[50] Hyun Y.H., Lim S.T., Choi H.J., Jhon M.S. (2001). Rheology of poly(ethylene oxide)/organoclay nanocomposites. Macromolecules.

[51] Cox W.P., Merz E.H. (1958). Correlation of dynamic and steady flow viscosities. J. Polym. Sci..

[52] Kim H., Abdala A.A., Macosko C.W. (2010). Graphene/polymer nanocomposites. Macromolecules.

[53] Rostami A., Nazockdast H., Karimi M. (2016). Graphene induced microstructural changes of PLA/MWCNT biodegradable nanocomposites: Rheological, morphological, thermal and electrical properties. RSC Adv..

[54] Gupta A., Simmons W., Schueneman G.T., Hylton D., Mintz E.A. (2017). Rheological and thermo-mechanical properties of poly(lactic acid)/lignin-coated cellulose nanocrystal composites. ACS Sustain. Chem. Eng..

[55] Kim Y.H., Kwon S.H., Choi H.J., Choi K., Kao N., Bhattacharya S.N., Gupta R.K. (2016). Thermal, mechanical, and rheological characterization of polylactic acid/halloysite nanotube nanocomposites. J. Macromol. Sci. B.

[56] Harrell E.R., Nakajima N. (1984). Modified Cole–Cole plot based on viscoelastic properties for characterizing molecular architecture of elastomers. J. Appl. Polym. Sci..

[57] Kashi S., Gupta R.K., Baum T., Kao N., Bhattacharya S.N. (2018). Phase transition and anomalous rheological behaviour of polylactide/graphene nanocomposites. Compos. B Eng..

[58] Nair S.T., Vijayan P.P., Xavier P., Bose S., George S.C., Thomas S. (2015). Selective localisation of multi walled carbon nanotubes in polypropylene/natural rubber blends to reduce the percolation threshold. Compos. Sci. Technol..

